# Beyond Nicotinamide Metabolism: Potential Role of Nicotinamide *N*-Methyltransferase as a Biomarker in Skin Cancers

**DOI:** 10.3390/cancers13194943

**Published:** 2021-09-30

**Authors:** Roberto Campagna, Valentina Pozzi, Davide Sartini, Eleonora Salvolini, Valerio Brisigotti, Elisa Molinelli, Anna Campanati, Annamaria Offidani, Monica Emanuelli

**Affiliations:** 1 Department of Clinical Sciences, Polytechnic University of Marche, 60100 Ancona, Italy; r.campagna@univpm.it (R.C.); v.pozzi@staff.univpm.it (V.P.); e.salvolini@univpm.it (E.S.); m.emanuelli@univpm.it (M.E.); 2 Department of Clinical and Molecular Sciences, Polytechnic University of Marche, 60100 Ancona, Italy; valeriobrisigotti@hotmail.it (V.B.); e.molinelli@pm.univpm.it (E.S.); a.campanati@univpm.it (A.C.); a.m.offidani@univpm.it (A.O.); 3 New York-Marche Structural Biology Center (NY-MaSBiC), Polytechnic University of Marche, 60131 Ancona, Italy

**Keywords:** skin cancer, melanoma, non-melanoma skin cancer, nicotinamide *N*-methyltransferase, biomarker

## Abstract

**Simple Summary:**

Skin cancers (SC) are a frequent type of malignancy in white populations and include malignant melanoma and non-melanoma skin cancer. Due to their increasing incidence rate worldwide, aggressive behavior, and usually late diagnosis, they represent an important challenge for health care systems. Therefore, identifying new biomarkers suitable for diagnosis, as well as for prognosis and targeted therapy is mandatory. Nicotinamide *N*-methyltransferase (NNMT) is an enzyme that plays a key role in the progression of several malignancies. There is increasing evidence that NNMT is also involved in the malignant behavior of SC. Therefore, this review aims to summarize the current state of the art regarding NNMT role in SC and to support future studies focused on exploring the diagnostic and prognostic potential of NNMT in skin malignancies, as well as its suitability for targeted therapy.

**Abstract:**

Skin cancers (SC) collectively represent the most common type of malignancy in white populations. SC includes two main forms: malignant melanoma and non-melanoma skin cancer (NMSC). NMSC includes different subtypes, namely, basal cell carcinoma (BCC), squamous cell carcinoma (SCC), Merkel cell carcinoma (MCC), and keratoacanthoma (KA), together with the two pre-neoplastic conditions Bowen disease (BD) and actinic keratosis (AK). Both malignant melanoma and NMSC are showing an increasing incidence rate worldwide, thus representing an important challenge for health care systems, also because, with some exceptions, SC are generally characterized by an aggressive behavior and are often diagnosed late. Thus, identifying new biomarkers suitable for diagnosis, as well as for prognosis and targeted therapy is mandatory. Nicotinamide *N*-methyltransferase (NNMT) is an enzyme that is emerging as a crucial player in the progression of several malignancies, while its substrate, nicotinamide, is known to exert chemopreventive effects. Since there is increasing evidence regarding the involvement of this enzyme in the malignant behavior of SC, the current review aims to summarize the state of the art as concerns NNMT role in SC and to support future studies focused on exploring the diagnostic and prognostic potential of NNMT in skin malignancies and its suitability for targeted therapy.

## 1. Introduction

Skin cancers (SC) are the neoplasms with the highest incidence in white populations, and their incidence has gradually intensified in the last decade [1,2]. The term “skin cancer” identifies two main forms, namely, malignant melanoma and non-melanoma skin cancer (NMSC). NMSCs include several subtypes, such as basal cell carcinoma (BCC), squamous cell carcinoma (SCC), Merkel cell carcinoma (MCC), and the two pre-neoplastic conditions Bowen disease (BD) and actinic keratosis (AK) [1,3].

Among all SC, malignant melanoma, that arises from altered pigment cells i.e., the melanocytes, is considered to be the most aggressive type [4]. In fact, while malignant melanoma represents only 1% of all SC, it is responsible for the majority of SC-related deaths [5]. Although the predominant part of malignant melanomas involves the skin, in particular, 25% of cutaneous melanomas affects the head and neck, the neoplasm may also arise in mucosal surfaces, the meninges, and the uveal tract [6]. Due to its aggressiveness, it is extremely important to diagnose malignant melanoma when it is in early stages, since the 5-year survival rate is 99% if the disease is diagnosed when still localized, while it drops to 27% if the disease is already metastatic at the time of diagnosis [5,7]. Several risk factors have been associated with malignant melanoma development such as a family history of SC, male sex, fair skin, amount of moles, and age, while the main environmental risk factor is ultraviolet (UV) exposure [7,8,9,10,11,12,13]. Indeed, cutaneous melanoma develops primarily in Caucasian people as the consequence of chronic sun exposure [14,15].

A diagnosis of malignant melanoma is facilitated by the ABCDEF criteria, which include lesion Asymmetry, Border irregularity, Color variegation, Diameter > 6 mm, Evolution of a nevus, and a nevus characteristic of Looking Funny, describing a malignant nevus that does not match in appearance with the other nevi variants displayed by a patient [16]. Upon diagnosis, the stage of malignant melanoma is identified considering the rules created by the American Joint Committee on Cancer (AJCC) to manage patient treatment and prognosis. Following these rules, melanomas are classified into five distinct stages, from 0 (melanoma in situ) to IV (metastatic melanoma), distinguished by a worsening prognosis [17]. A variation of the classical TNM system is used by AJCC criteria to characterize melanoma (from early-stage to late-stage) by analyzing the tumor thickness with or without ulceration, nodal involvement, and presence of metastasis [17]. Once diagnosed, surgical resection represents the best opportunity for the definitive cure of a primary melanoma. Other therapeutical options include radiotherapy, chemotherapy, immunotherapy, and targeted therapies [7,18,19,20,21,22,23]. However, while surgery can achieve 99% of success if the diagnosed melanoma is in situ, melanomas at advanced stages are problematic to be treated [24]. This occurs due to the presence of metastases (lymph nodes, lungs, brain, liver, and bone are the most frequent metastatic sites), to an intrinsic resistance towards most of the therapies available nowadays, and to the high genomic heterogeneity that characterizes melanocytic tumors [25]. In this regard, the identification of novel biomarkers that could be used as prognostic or predictive markers and as objectives of targeted therapies is of utmost importance. Although, in the last years, several biomarkers have been proposed (e.g., microphthalmia-associated transcription factor, cyclooxygenase-2, chondroitin sulfate proteoglycan 4, human melanoma black-45), none of them has become of routine use in the clinical practice, with the exception of BRAF and MEK that are targets of specific inhibitors used with success in the clinical practice, but to only a small subset of patients responds [26].

BCCs arise from basal keratinocytes and account for approximately 75–80% of NMSCs but they are characterized by a more benign behavior, having a very limited metastatic potential [27]. However, it is the most common malignancy in humans, and several histological subtypes have been described, each of them characterized by different clinical features, outcomes, and prognosis [28,29]. The nodular subtype is the most common type, distinguished by the presence of large nodules of tumor cells within the dermis, and represents a low-risk type.

On the contrary, infiltrating BCC is a more aggressive variant, consisting of narrow tumor cords and nests of atypical basaloid cells with an infiltrative growth pattern. Since it displays a high risk of recurrence, this variant requires a more careful approach with an accurate evaluation of its surgical margins [3]. Regardless of the subtype, due to the fact that aging is one the main risk factors, and given that the global population age is increasing, an increase in its related morbidity and local recurrence rates is expected [30].

SCC develops from stratum spinosum keratinocytes and is classified as well, moderately and poorly differentiated. Analogously to BCC, the increase in the average population age and in the exposure to UV light is resulting in an increase in SCC diagnosis [31,32]. SCC occurs often in the head and neck region, where it emerges as an erythematous scaling nodule and plaque, eventually ulcerated. SCC can be classified in several subtypes that differ for their histological appearance and prognosis. The most common subtype displays atypical keratinocytes invading the dermis [33,34]. Depth of invasion (tumor thickness >2 mm), tumor size (diameter >2 cm), acantholysis, perineural and vascular involvement are considered negative prognostic factors. Furthermore, an association between the site of the primary tumor and prognosis was demonstrated, since head and neck SCCs are more prone to metastasize compared to tumors that arises on the extremities or trunk [35,36]. Indeed, despite the fact that surgical excision is curative for most of SCCs, a subset of patients will undergo relapse and eventually will develop metastasis. Hence, the identification of biomarkers with a prognostic value that could support SCC treatment and promote an adjuvant targeted therapy is a primary goal.

Keratoacanthoma (KA) is a malignancy displaying a bi-phasic growth pattern characterized by a fast growing phase generally followed by involution. As most of the other SCs, UV exposure is a risk factor, since KA mostly occurs on sun-damaged skin [37]. There is not a consensus among authors on whether KA is a variant of SCC or a separate entity; however, a certain differential diagnosis between these two malignancies is of primary need, since KA, unlike SCC, is characterized by a good prognosis due to its inclination to spontaneous involution [37,38].

As concerns BD, it is believed to be an in situ SCC, whereas AK can be considered a pre-cancerous lesion which may develop in SCC. Although both diseases exhibit a close association with SCC, they are characterized by different histopathological characteristics [39].

MCC is usually present as erythematous nodule characterized by rapid growth. In the past, MCC was considered to derive from skin Merkel cells, and this explains its name. However, nowadays it is believed that MCC arises from skin precursors of epithelial, lymphoid, or fibroblastic type. Although it represents <1% of all NMSCs, it displays a very aggressive behavior reflected by the presence of clinical or pathological node disease in up to 48% of the patients at diagnosis, while 10% of them already display a metastatic stage at diagnosis [40]. The combination of surgery and radiotherapy is considered the first line of treatment; nonetheless, since recurrence rates are high, about 40% of patients will undergo recurrence within 2 years of diagnosis [41].

Since early and accurate diagnosis and prognosis have a crucial impact on the outcome of these diseases, clinical practice is constantly looking for new genetic and molecular markers that could facilitate an early diagnosis or an accurate setting of the prognosis for both cancerous and non-cancerous diseases, in order to reduce morbidity and improve patients’ survival [30,42,43,44,45,46]. This is particularly relevant for SC, since the number of new cases is expected greatly increase in the next future due to increasing UV exposure and population age [1,47,48].

## 2. Nicotinamide (NAM) in SC

NAM is a form of vitamin B3 largely utilized for the management of several chronic dermatoses, which includes rosacea, acne, blistering immune disorders, atopic dermatitis, and cutaneous neoplasms [49]. NAM is the precursor of nicotinamide adenine dinucleotide (NAD^+^), a co-enzyme of redox reactions crucial for the production of adenosine triphosphate (ATP). For this reason, it is a master influencer of cellular metabolism, regulating multiple pathways involved in both cellular survival and apoptosis [50].

UV exposure induces damage of cellular DNA, triggering strand breaks, crosslinks, and base modifications that are repaired by various repair systems, which in turn consume ATP [51,52]. Therefore, UV exposure leads to ATP consumption, which in turn induces a kind of cellular energy crisis, and, since repair systems need high levels of ATP to work properly, the consequence is an accumulation of molecular aberrations and genome instability [53]. Therefore, it was reported that NAM exerts UV protective effects due its involvement in cellular energy pathways, as a precursor of NAD^+^ [54]. In detail, UV irradiation causes the block of glycolysis by activating poly-ADP-ribose-polymerase 1 (PARP-1), and this event ultimately inhibits NAD^+^ production [55,56]. Furthermore, NAM inhibits skin carcinogenesis regulating the proteins p53 and sirtuins. Indeed, when DNA damage is too extensive, p53 is activated by NAD^+^ and triggers cell cycle arrest and apoptosis. Since NAM is able to restore the intracellular levels of NAD^+^, it was reported that NAM can modulate the p53 pathway [57]. Moreover, NAM was proposed to be a negative regulator of SIRT1, a NAD^+^-dependent histone deacetylase able to inhibit p53, thus preventing apoptosis [58,59].

NAM was demonstrated to play a key role in preventing skin carcinogenesis by counteracting UV-induced immunosuppression. Indeed, UVB-induced DNA damage stimulates cutaneous antigen-presenting cells (APC) to produce interleukin (IL)-10, which downregulates the immune response [60]. According to this hypothesis, the topic use of NAM in UV-irradiated mice could counteract the cutaneous carcinogenesis process. NAM supplementation through the diet was able to decrease SC incidence in UV-exposed mice with a dose-dependent effect [61]. Another study demonstrated that a group of people taking oral nicotinamide had a minor diminution of delayed-type hypersensitivity when exposed to UV light for three consecutive days compared to the placebo group. The protective effect of NAM was ascribable to its activity of counteracting immunosuppression by restoring the sufficient energy levels demanded by cells for repairing DNA damage and preventing PARP overactivation [62].

In order to evaluate the chemopreventive effect of NAM in high-risk patients, the Phase III double-blind ONTRAC Study was designed [63]. In this study, patients were selected for having had two or more NMSC in the last 5 years. In the period of treatment, which lasted 12 months, the mean incidence of NMSC observed in the nicotinamide-treated group was 1.8, while that in the placebo group was 2.4. In particular, the mean number of BCCs was 1.3 in the nicotinamide-treated group and 1.9 in the placebo group, with a smaller rate of 20% after adjustment for medical center of treatment and 5-year BCC history. The mean number of SCCs was 0.5 in the nicotinamide-treated group and 0.7 in the placebo group, with a smaller rate of 30% after adjustment for center and 5-year SCC history [63].

As regards malignant melanoma, studies performed in vitro demonstrated that NAM is able to improve the repair rate of the nucleotide excision system and can increase the percentage of melanocytes undergoing DNA repair after UV exposure [64]. Subsequent in vitro studies on melanoma cell lines demonstrated that NAM supplementation, equal to the orally administered doses utilized in the ONTRAC study, did not boost cell viability, proliferation, or invasiveness. Nonetheless, NAM was able to induce an immune response directed to the existing melanomas in vivo [65]. Taken together, these findings led to the hypothesis that oral NAM does not worsen melanoma pathogenesis but, on the contrary, might be useful in melanoma chemoprevention.

## 3. Nicotinamide *N*-Methyltransferase

Nicotinamide *N*-methyltransferase (NNMT) is an enzyme that catalyzes the *N*-methylation of nicotinamide, using *S*-adenosyl-l-methionine (SAM) as a methyl donor, thus yielding *N*1-methylnicotinamide (MNA) as a product and releasing *S*-adenosyl-l-homocysteine (SAH) [66]. Since it can also methylate other pyridines and other structural analogs, it plays a pivotal role not only in nicotinamide homeostasis but also in the biotransformation and detoxification of several xenobiotic compounds [67,68]. Furthermore, it was demonstrated that NNMT takes part also in several crucial metabolic pathways. 

**Figure 1:**
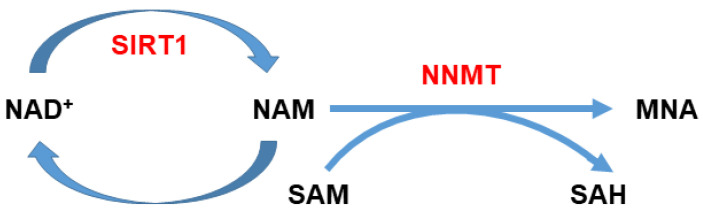
Nicotinamide (NAM) metabolism. NAM can be methylated by nicotinamide *N*-methyltransferase (NNMT) utilizing *S*-adenosyl-l-methionine (SAM) as a methyl donor, which in turn is converted to *S*-adenosyl-l-homocysteine (SAH). NNMT activity can affect NAD^+^ biosynthesis and thus ATP production, since it can regulate the amount of NAM converted into NAD^+^. Furthermore, by modulating the intracellular SAM/SAH ratio, it can indirectly impact gene expression.

Nicotinamide is the precursor of nicotinamide adenine dinucleotide (NAD^+^), a co-enzyme of redox reactions required for ATP production. Therefore, the amount of nicotinamide inside the cell available for energy metabolism can be regulated by NNMT activity (Figure 1), and therefore, the catalytic activity of the enzyme can affect and modulate multiple pathways of cellular survival and apoptosis [50]. In addition, by influencing the SAM/SAH ratio inside the cell, it can indirectly impact gene expression [69]. In the last two decades, NNMT has been the focus of a number of studies that demonstrated the involvement of this enzyme in the progression of numerous malignancies including oral squamous cell carcinoma (OSCC), papillary thyroid cancer, lung cancer, gastric cancer, pancreatic cancer, colorectal cancer, clear cell renal cell carcinoma (ccRCC), breast cancer, bladder urothelial carcinoma (BUC), and ovarian clear cell carcinoma [70,71,72,73,74,75,76,77,78,79,80,81,82,83,84,85].

The analysis of NNMT expression levels in ccRCC demonstrated that the amount of upregulated enzyme is inversely correlated with tumor size, suggesting that the enzyme could play a role in cancer progression [86]. Similar results were obtained in OSCC, for which NNMT upregulation was negatively correlated with the parameters pT, lymph node metastasis, pathological and histological grading; this evidence led to hypothesize its potential involvement in tumor growth and differentiation [87,88].

NNMT expression levels were also found to be notably upregulated in exfoliated cells isolated from the urine of BUC patients compared to that of controls, and an inverse correlation between enzyme expression and histological grade was demonstrated, an observation that suggested the remarkable diagnostic accuracy of a urine test based on the detection of NNMT levels [83]. Consistently with these findings, an increased level of NNMT was detected in saliva samples of OSCC patients compared to controls, a finding that suggested the use of NNMT as a salivary biomarker for the early and non-invasive diagnosis of oral cancer [89]. Taken together, these studies demonstrate that the NNMT enzyme has a remarkable potential as a diagnostic and prognostic biomarker in a wide spectrum of malignancies.

## 4. Involvement of NNMT in SC

Given the increasing evidence that NNMT relevantly contributes to cancer progression, several studies have been performed in order to explore its potential involvement also in SC. A summary of the results of all studies is reported in Table 1.

Ganzetti et al. were the first authors to investigate the role of NNMT in malignant melanoma. In this retrospective study, a total of 34 primary melanomas and 34 melanocytic non-congenital non-atypical compound and dermal nevi, used as the control group, were analyzed by immunohistochemistry. In this work, a significantly higher NNMT expression level was found in cutaneous malignant melanoma samples compared to benign nevi [90]. An analysis of NNMT expression in melanoma samples from the Pan-Cancer Analysis of Whole Genomes (PCAWG) (https://www.ebi.ac.uk/gxa/home) (accessed on 14 September 2021) confirmed these findings, showing that the enzyme displayed an expression level of 26 transcript per million (TPM), while in other types of cancers, characterized by a marked overexpression of NNMT, such as bladder, lung, and breast cancer, an expression level of 21, 161, and 71 TPM, respectively, was detected. These results demonstrate that NNMT overexpression in melanoma is remarkable. Furthermore, Ganzetti et al. demonstrated that the NNMT levels measured in the melanoma samples resulted to be inversely correlated to Breslow thickness, Clark level, the presence and number of mitoses, and ulceration, thus suggesting that the enzyme has a good potential to be used as a prognostic biomarker [90]. A subsequent immunohistochemical study regarding NNMT expression levels in cutaneous melanoma confirmed these findings [91]. In the same study, the authors also analyzed the enzyme expression level in samples of patients with oral malignant melanoma, an exceptionally rare and aggressive variant of the neoplasm of the head and neck region, which notoriously displays a poor prognosis. The immunohistochemical analysis revealed that NNMT expression was significantly higher in cutaneous malignant melanoma samples, but oral malignant melanoma samples exhibited more strongly stained cells, thus suggesting a potential involvement of NNMT in oral malignant melanoma. Furthermore, the findings presented in this work indicated that NNMT levels, measured in both oral malignant melanoma and cutaneous melanoma samples, showed a potential association with the presence of ulcers, which was contrasting in the two neoplasms, since the staining intensity was higher in ulcerated oral malignant melanoma samples, while the ulcerated cases of cutaneous melanoma displayed a reduction of NNMT levels. Finally, statistical analysis revealed an inverse correlation between the percentage of NNMT-positive cells in the tumor samples and the disease-free survival time in oral malignant melanoma patients, indicating that NNMT could be an efficient prognostic factor for this malignancy [91]. 

In another study, the functional role of NNMT was investigated through shRNA-mediated silencing of the enzyme in the melanoma cell lines A375 and WM-115 [92]. Following NNMT knockdown, cell proliferation, migration, and chemosensitivity were evaluated. The data obtained revealed that enzyme silencing triggered a significant reduction of cell proliferation and migration in A375 melanoma cells. Furthermore, enzyme downregulation sensitized melanoma cells to the chemotherapeutic dacarbazine. In addition, similar effects on cell proliferation and chemosensitivity were obtained in WM-115 melanoma cells, upon enzyme silencing. These findings led to the hypothesis that NNMT might be involved in promoting mechanisms of chemoresistance. A subsequent study explored the role of NNMT also in NMSC. A total of 79 specimens (40 BCC and 39 SCC cases) were subjected to immunohistochemical analysis to evaluate the enzyme expression levels, with the healthy tissue margins used as a control [94]. In the BCC cohort, NNMT expression was significantly higher in tumor specimens than in normal tissue margins. Interestingly, immunopositivity was higher in nodular BCC compared to the infiltrative BCC subtype. This expression pattern was also exhibited by BCCs displaying both nodular and infiltrative features within a single tumor. Therefore, the obtained data suggest an inverse correlation between NNMT expression and tumor aggressiveness [94].

Regarding SCC, the study analyzed both tumor samples from the head and neck region and lesions affecting the rest of the body. Unexpectedly, the findings reported in this study showed a significant lower NNMT expression in cancer cells compared to healthy margin tissues. Interestingly, the fraction of immuno-positive cells was markedly higher in SCC specimens excised from extremities and trunk compared to specimens from the head and neck, thus reinforcing the hypothesis of an inverse correlation between enzyme expression and tumor aggressiveness [94]. Altogether, these findings suggest that NNMT may be a potential prognostic biomarker for these neoplasms.

A subsequent study analyzed differences in protein expression between the human skin squamous carcinoma cell lines SCC12 and SCC13, with the aim to identify which genes determine the high invasive potential displayed by the SCC12 cell line compared with the poorly invasive SCC13 cell line [93].

Using matrix-assisted laser desorption/ionization time-of-flight mass spectrometry (MALDI–TOF-MS), the authors identified NNMT as an upregulated protein in the SCC12 cell line. Therefore, shRNA silencing of the enzyme was performed in order to evaluate the impact of NNMT knockdown on cell proliferation, migration, and invasion. NNMT downregulation strongly inhibited the proliferation and density-dependent growth of SCC cells, as well as their migration and invasion. Furthermore, the impact of NNMT knockdown on epithelial–mesenchymal transition (EMT)-associated gene expression was investigated through the RT^2^ Profiler PCR Array. The results showed that NNMT silencing was able to downregulate 10 of the 84 EMT-related genes analyzed, namely the genes coding for MMP9, SPP1, and versican core protein (VCAN), which play a role in the modulation of extracellular matrix (ECM) structure and function. Furthermore, the mRNA expression level of Slug, a key effector of EMT, was also repressed by NNMT silencing. In the light of the above-mentioned findings, NNMT was proposed as a novel prognostic biomarker and therapeutic target for patients with SCC [93].

Another study evaluated the differential expression of NNMT in cutaneous KA and SCC on 48 samples through immunohistochemistry [95]. The reported results demonstrated a significantly higher NNMT expression level in KA compared to SCC. In detail, the percentage of NNMT-positive cells was significantly lower in head and neck SCC compared to SCC samples from the rest of the body. It is noteworthy that, according to previous studies, tumors with a less favorable prognosis displayed reduced NNMT levels [86,87,90,94]. Since KA and well-differentiated SCC are difficult to be distinguished from a histopathological perspective, the observed differences in NNMT expression may be exploited to perform a prompt differential diagnosis between these two pathological conditions. Indeed, while KA is characterized by an excellent prognosis due to its natural tendency to involute, SCC is characterized by a very aggressive behavior. Therefore, these findings reinforce the idea that NNMT may be a novel biomarker suitable for both the early diagnosis and prognosis of these neoplasms, and for designing targeted therapeutic strategies.

## 5. Conclusions and Future Perspectives

Even though the contribution of NNMT to the cancer progression was demonstrated in a large number of malignancies, the effective role of this enzyme in cancer cells still needs to be fully elucidated.

The above-mentioned studies demonstrated that NAM exerts a positive role in counteracting carcinogenesis. On the other hand, the enzyme NNMT, the master regulator of intracellular NAM, seems to be involved in tumor progression. Notably, given the chemopreventive role of NAM, it is conceivable that NNMT may exert a primary role in the first step of carcinogenesis, irreversibly methylating NAM, thus generating MNA. Since overexpression of NNMT was reported in most SC, the enzyme activity may determine a drop in the intracellular levels of NAM, resulting in UV sensitization of cells and impairing the mechanisms involved in cycle cell arrest and DNA repair, as discussed above. All these events may be responsible for neoplastic cell transformation over time. In this regards, further studies are required in order to elucidate whether NNMT overexpression is responsible for the neoplastic transformation of cells or whether it is a consequence of the altered gene expression pattern of the neoplastic cell.

Nevertheless, it clearly appears that NNMT might be an excellent candidate as a diagnostic and prognostic marker in skin cancers. The studies performed to date are promising, but further analyses are required in order to widen the cohort of patients taken into consideration, thus confirming the suitability of the enzyme as a biomarker in the clinical practice.

A large number of studies were focused on exploring the impact of NNMT downregulation in several cancer models, leading to the discovery that the suppression of this enzyme prevents cancer cell proliferation, invasion, and metastasis, as well as chemoresistance [50,92]. Moreover, it was suggested that the enzyme may contribute to the radioresistance of cancer cells [96,97]. In the light of the above-mentioned considerations, NNMT can also be considered a promising molecule for targeted therapy.

One of the encouraging frontiers that has recently drawn much attention is the development of specific inhibitors of the enzyme, which are providing encouraging results [98,99,100,101,102,103]. It remains to be seen whether the strong preclinical evidence of small-molecule inhibitors against NNMT could still be translated in clinical practice for patients’ treatment. Therefore, appropriate studies should be performed in this direction. 

## Figures and Tables

**Table 1:** Summary of studies exploring the role of NNMT in SC.

Type of Skin Cancer	Diagnostic Potential	Prognostic Potential	Therapeutical Target	Reference
Cutaneous malignant melanoma	Yes	Yes (inverse correlation)	N.A.	[89]
Cutaneous malignant melanoma	Yes	Yes (inverse correlation)	N.A.	[90]
Oral malignant melanoma	No	Yes (positive correlation)	N.A.	[90]
Human malignant melanoma cell lines	N.A.	N.A.	Yes	[91]
Basal cell carcinoma	Yes	Yes (inverse correlation)	N.A.	[92]
Squamous cell carcinoma	No	Yes (inverse correlation)	N.A.	[92]
Keratoacanthoma	Yes	Yes (inverse correlation)	N.A.	[93]
Human skin squamous carcinoma cell lines SCC12/13	N.A.	Yes	N.A.	[94]
